# Cell Origin of Human Mesenchymal Stem Cells Determines a Different Healing Performance in Cardiac Regeneration

**DOI:** 10.1371/journal.pone.0015652

**Published:** 2011-02-10

**Authors:** Ralf Gaebel, Dario Furlani, Heiko Sorg, Bianca Polchow, Johannes Frank, Karen Bieback, Weiwei Wang, Christian Klopsch, Lee-Lee Ong, Wenzhong Li, Nan Ma, Gustav Steinhoff

**Affiliations:** 1 Reference and Translation Center for Cardiac Stem Cell Therapy (RTC), Department of Cardiac Surgery, University of Rostock, Rostock, Germany; 2 Institute of Transfusion Medicine and Immunology, German Red Cross Blood Service of Baden-Württemberg-Hessen, Mannheim, Germany; University of Bristol, United Kingdom

## Abstract

The possible different therapeutic efficacy of human mesenchymal stem cells (hMSC) derived from umbilical cord blood (CB), adipose tissue (AT) or bone marrow (BM) for the treatment of myocardial infarction (MI) remains unexplored. This study was to assess the regenerative potential of hMSC from different origins and to evaluate the role of CD105 in cardiac regeneration. Male *SCID* mice underwent LAD-ligation and received the respective cell type (400.000/per animal) intramyocardially. Six weeks post infarction, cardiac catheterization showed significant preservation of left ventricular functions in BM and CD105^+^-CB treated groups compared to CB and nontreated MI group (MI-C). Cell survival analyzed by quantitative real time PCR for human GAPDH and capillary density measured by immunostaining showed consistent results. Furthermore, cardiac remodeling can be significantly attenuated by BM-hMSC compared to MI-C. Under hypoxic conditions *in vitro*, remarkably increased extracellular acidification and apoptosis has been detected from CB-hMSC compared to BM and CD105 purified CB-derived hMSC. Our findings suggests that hMSC originating from different sources showed a different healing performance in cardiac regeneration and CD105^+^ hMSC exhibited a favorable survival pattern in infarcted hearts, which translates into a more robust preservation of cardiac function.

## Introduction

Cell transplantation utilizing different cell types including skeletal myoblasts [1], [2], cardiomyocytes [3], [4], smooth muscle cells [5], [6], bone marrow cells [7] and hematopoietic stem cells [8], has emerged as a promising therapeutic avenue for cardiac regeneration following myocardial infarction damage. Experimental and clinical evidences have demonstrated that transplanting different cell types in the myocardium is safe and could contribute to the angiogenesis in the infarcted area and the improvement of cardiac functions [9]–[14].

Among the various cell types investigated, human mesenchymal stem cells (hMSC) derived from adult tissues are an attractive stem cell source in cardiac regeneration. hMSC are multipotent [15] and capable of differentiating into cardiomyocytes [16] under appropriate conditions. They express genes encoding for anti-apoptotic, angio-/arteriogenic factors [17] and vascular endothelial growth factor [18], and matrix-mediating factors [19]. Furthermore, hMSC can be easily isolated by plastic adherence from bone marrow (BM), umbilical cord blood (UCB) or adipose tissue (AT), and are culture expandable for therapeutic application. hMSC derived from different sources have been well characterized with respect to the isolation procedure, the cell number, multilineage differentiation capacity [20]–[28], and aging behavior of hMSC [29]–[32].

Wagner et al. evaluated hMSC from AT, UCB and BM in terms of immune phenotype with a panel of 22 surface antigen markers and analyzed their global gene expression profiles [33]. hMSC populations from different sources displayed similar phenotypic characteristics and a consistent and reproducible gene expression profile [33]. Kern et al. characterized the hMSC pertaining to their morphology, frequency of colonies, expansion characteristics, multilineage differentiation capacity, immunophenotype, and success rate of isolating the cells under identical in vitro conditions [34]. Although phenotypically similar, these culture-expanded hMSC exhibited cell source-related heterogeneity in colony frequency, proliferative potential and differentiation potential [34], suggesting these different sources might greatly affect the survivability and behavior of transplanted hMSC in the hostile environment of ischemia, inflammation, pro-apoptotic factors and scarring from myocardial infarction [35].

In this study we compared the therapeutic potential of hMSC dervied from different sources in cardiac regeneration in a SCID mouse left anterior descending (LAD) ligation model via intracardiac injection with respect to cell survival, infarct size, angiogenesis, cardiac remodelling and fuctional improvement. We found that CD105^+^ hMSC exhibited a favorable survival pattern in infarcted hearts, which translated into a more robust preservation of cardiac function.

## Results

### Immunophenotypic analysis and functional differentiation

**Figure 1:**
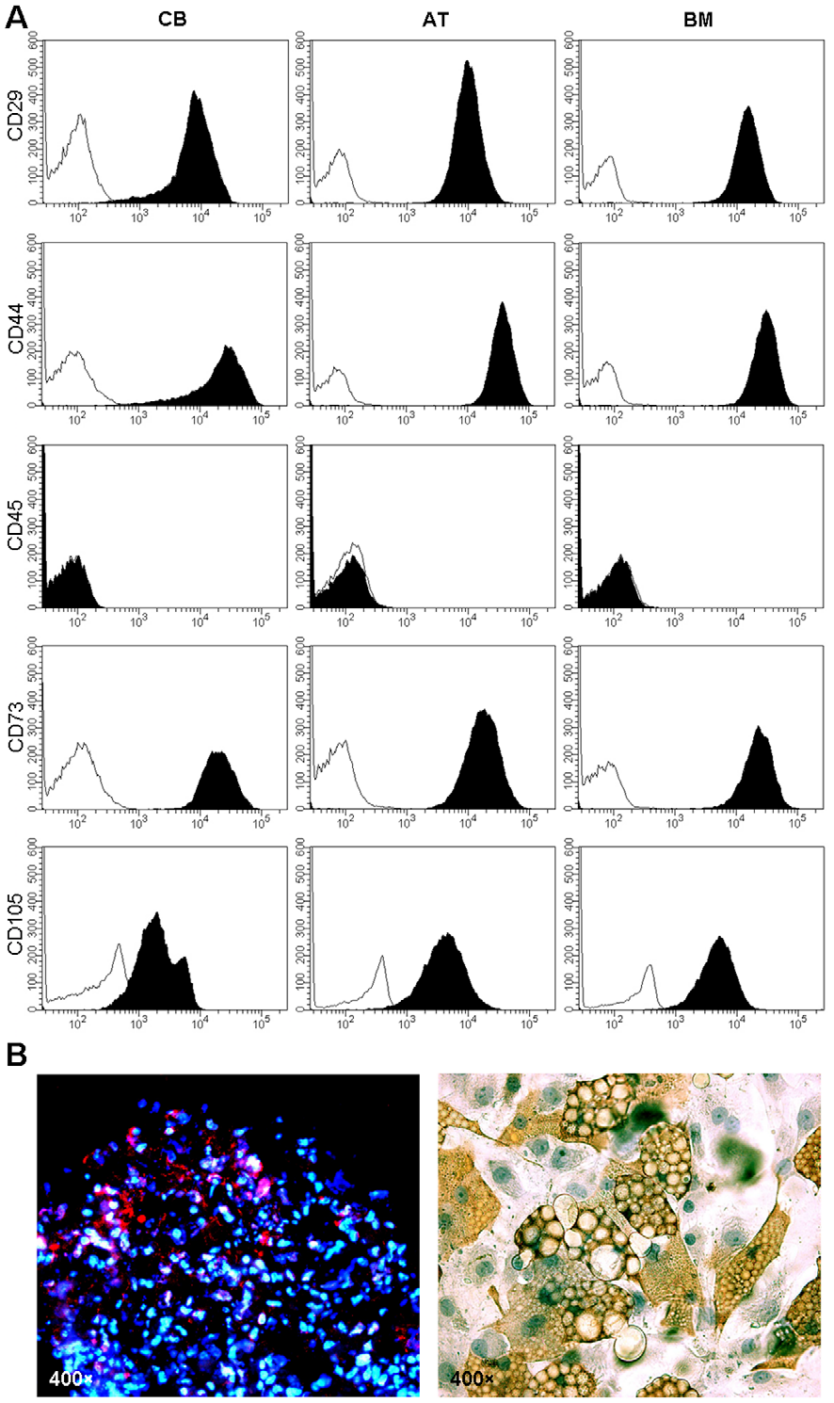
Phenotypic characterization of hMSC from different sources. ***A.*** FACS analysis showed that the cells were negative for CD45 expression and positive for CD29, CD44, CD73 and CD105, which are phenotypes currently known to be characteristic of hMSC. The gray line indicates the control of the CD marker isotypes. ***B.*** *In vitro* differentiation capacity of transplanted hMSC. hMSC from bone marrow were cultured in adipogenic and chondrogenic medium. Chondrogenic differentiation (left). Immunostaining for aggrecan (red). Nuclei were counterstained with DAPI (blue). Adipogenic differentiation (right). Immunostaining with fatty acid binding protein-4 (brown).

Cell isolation, expansion, characterization, and differentiation of hMSC have been established according to previous reports [23], [33]. The morphology of hMSC from different sources displayed a homogenous spindle-shaped population and maintained a similar morphology during the subsequent passages. FACS analysis was employed to identify the surface marker expression at passage 3. The hMSC culture was shown to be devoid of CD45, which is one of the markers for hematopoietic cells. In contrast, a high expression of CD29, CD44, CD73 and CD105 markers were observed from three different sources (Figure 1A). Compared to AT- and BM-hMSC, CD105 expression level in CB-hMSC was significantly lower (Figure 1A and Table 1). The results from FACS analysis were confirmed by immunocytochemistry (data not shown).

**Table 1:** Immunophenotypic analysis of hMSC from different origins.

Antibody	CB	AT	BM
CD29	99,5±0,3	99,4±0,1	98,3±0,6
CD44	98,8±0,4	99,2±0,1	98,0±0,4
CD45	0,9±0,1	0,6±0,1	0,5±0,2
CD73	99,9±0,0	99,2±0,1	98,9±0,1
CD90	99,7±0,1	95,2±0,8	95,9±1,6
CD105	66,9±0,8*	90,0±5,2	97,3±1,0

n = 3; mean [%]±SEM.

*p<0,05 compared to AT, BM.

### CD105^+^ isolation

For characterization of isolated CD105^+^ cells from human CB-derived hMSC compared to unseparated CB group, surface protein expression of 4 donors immediately after magnetic separation were examined by flow cytometry. A high percentage of cells express CD105 antigen with a mean value over 90% after magnetic isolation whereas a smaller population below 80% express CD105 antigen in unseparated CB group as previously described by Kern et al [34] (Figure 1A). To observe the CD105 antigen presence in later passages of isolated CD105^+^ cells, we examined CD105 surface protein expression of 3 donors after passage 4 and 5. During the growth of the cells in culture the CD105 surface marker decreased over the culture time (Table 2).

**Table 2:** CD105 expression in CB105^+^ hMSC over the culture time.

Antibody	CB105^+^ P3	CB105^+^ P4	CB105^+^ P5
CD105	99,0±0,2	80,3±1,0	59,6±1,0

n = 3; mean [%]±SEM.

hMSC can differentiate into multiple lineages (such as bone, cartilage, and adipose tissue), and this ability is taken as a functional criterion defining hMSC precursor cells [36]. To verify whether the CD105 enrichment affected the differentiation capacity, the hMSC from different sources and CD105 MSC-CB underwent adipogenic, and chondrogenic differentiation using the methods previously described [37], [38] at 24 hours post-enrichment. After 21 days of induction toward an adipogenic lineage, a characteristic morphological change with accumulation of lipid vacuoles was observed (Figure 1B left). Immunostaining revealed the presence of FABP-4, which is a marker protein for adipocytes. Chondrogenesis was assessed by immunostaining for aggrecan after 4 weeks of culture under chondrogenic conditions. The chondrocyte-like cells showed positive staining for aggrecan protein (Figure 1B right). Taken together, this evidence indicated that hMSC prior to in vivo experiment retain their multidifferentiation potential into adipogenic, and chondrogenic, lineages.

### Cardiac functions

**Figure 2:**
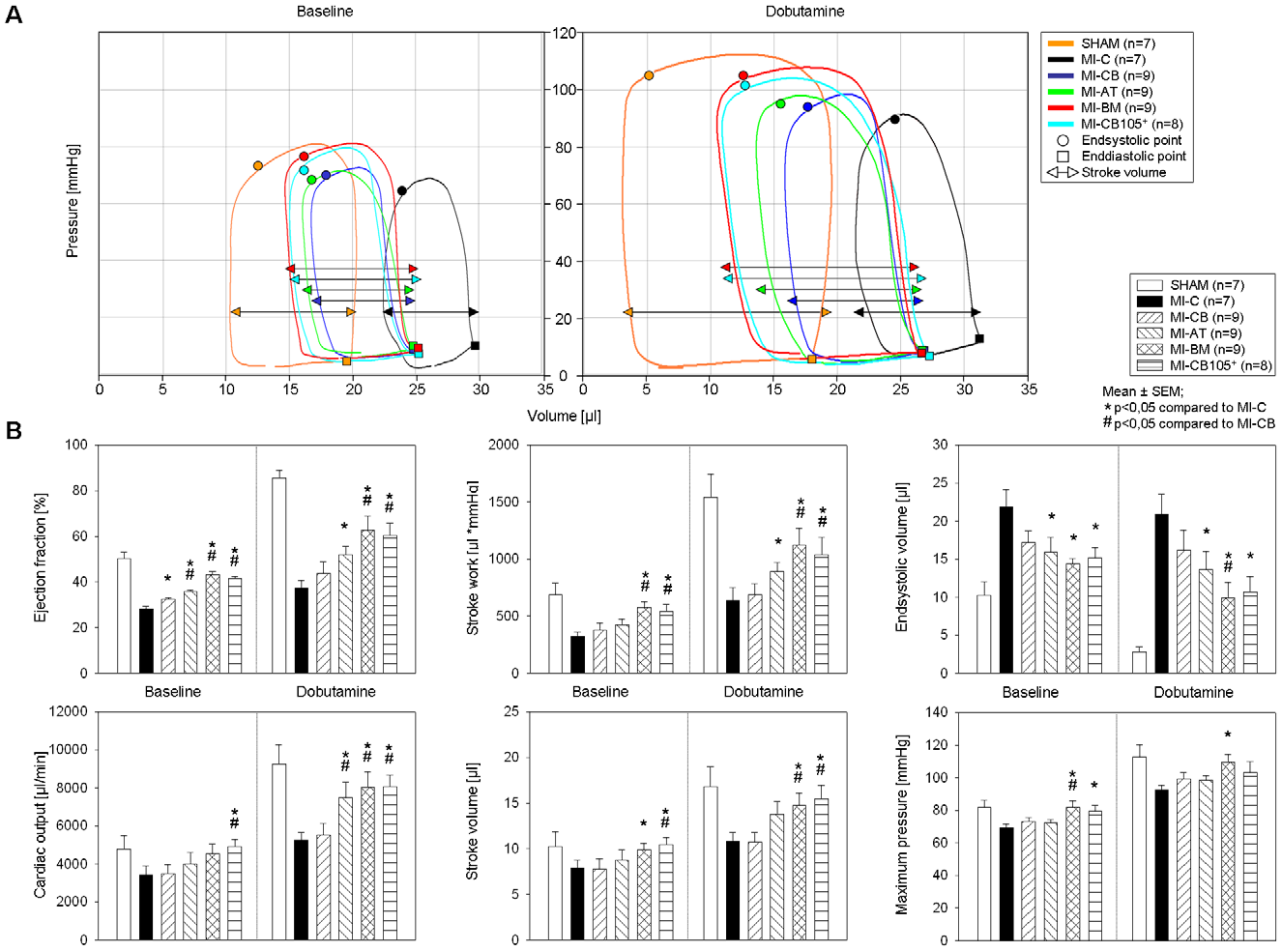
Heart functions 6 weeks after MI. ***A.*** Recovery of cardiac performance shows improvement for hearts with implanted human BM- and CD105-purified CB-hMSC compared to MI-CB hearts. ***B.*** Left ventricular functions at both baseline and stress condition assessed by catheterization.

Hemodynamic measurement of the cardiac performance (Figure 2A) demonstrates an improvement of functional parameters in case of stem cell treatment both under baseline conditions as well as after stress induction. Figure 2A also shows improved endsystolic values and stroke volume for hearts with implanted human BM- and CD105-purified CB-hMSC (MI-CB105) compared to MI-CB. Functional parameters in MI-BM and MI-CD105 groups present significant improvements on ejection fraction (EF) and stroke work (SW), both under baseline and stress induction in comparison to the MI-CB (Figure 2B). Significant values have also been found on stroke volume (SV), endsystolic volume (ESV), maximum pressure (Pmax) and cardiac output (CO; Figure 2B).

### Infarct size

**Figure 3:**
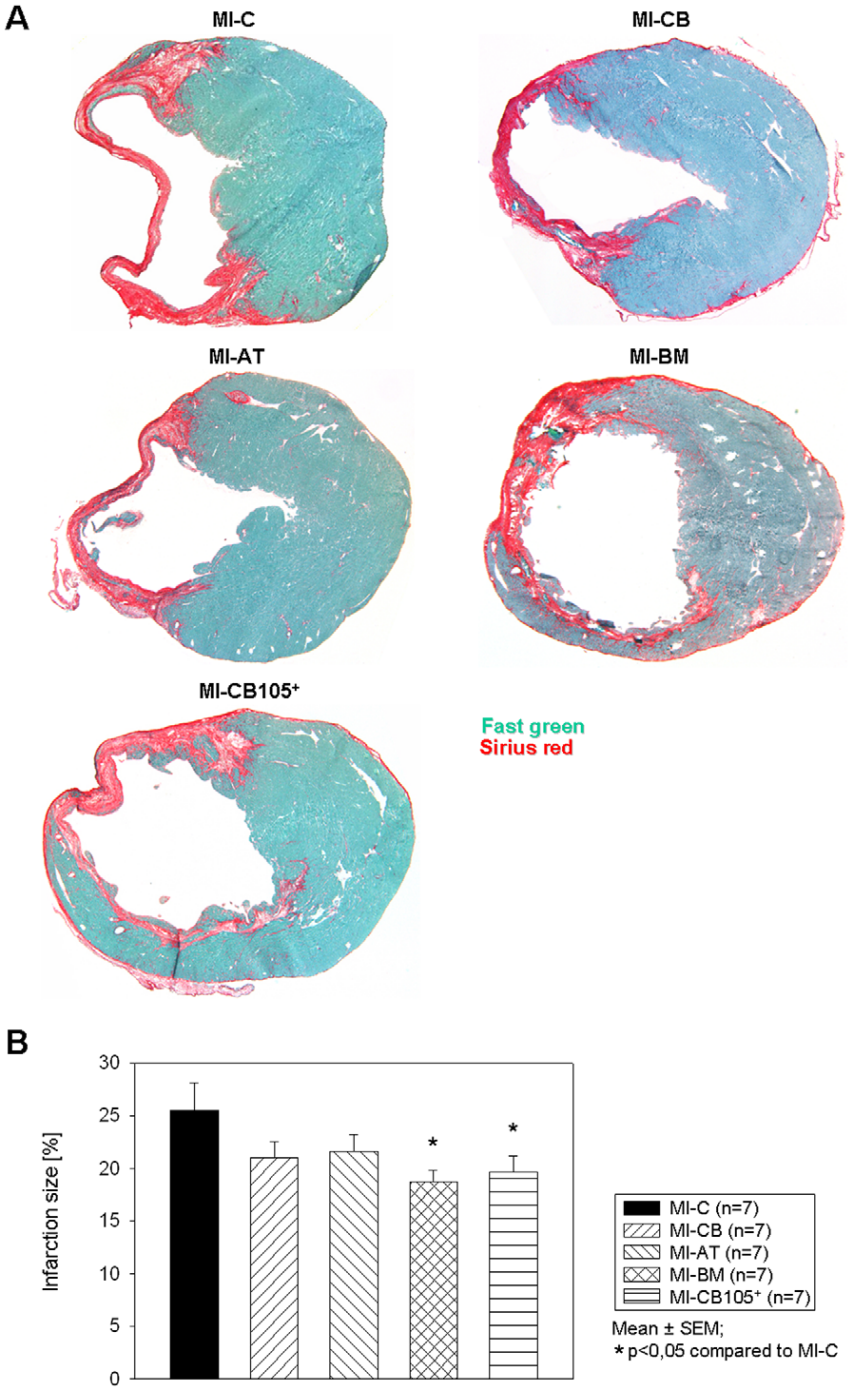
Infarction size 6 weeks after MI. ***A.*** Representative ventricular cross sections of heart level c. ***B.*** Ratio of infarction size to entire LV is significantly decreased in MI-BM and MI-CB105^+^ compared to MIC.

Ligation of the LAD consistently resulted in a transmural MI with its typical histologic changes including the thinning of the left ventricular free wall (Fast green) and extensive collagen deposition (Sirius red) 6 weeks after infarction. Representative heart sections 6 weeks after myocardial infarction following hMSC application or no injection of cells in the MI-C group are shown in Figure 3A. All hMSC treated animals show a markedly smaller infarct size, while the application of human BM- and CD105-purified CB-hMSC could significantly reduce (p<0.05) the myocardial damage compared to MI-C (Figure 3B).

### Capillary density

**Figure 4:**
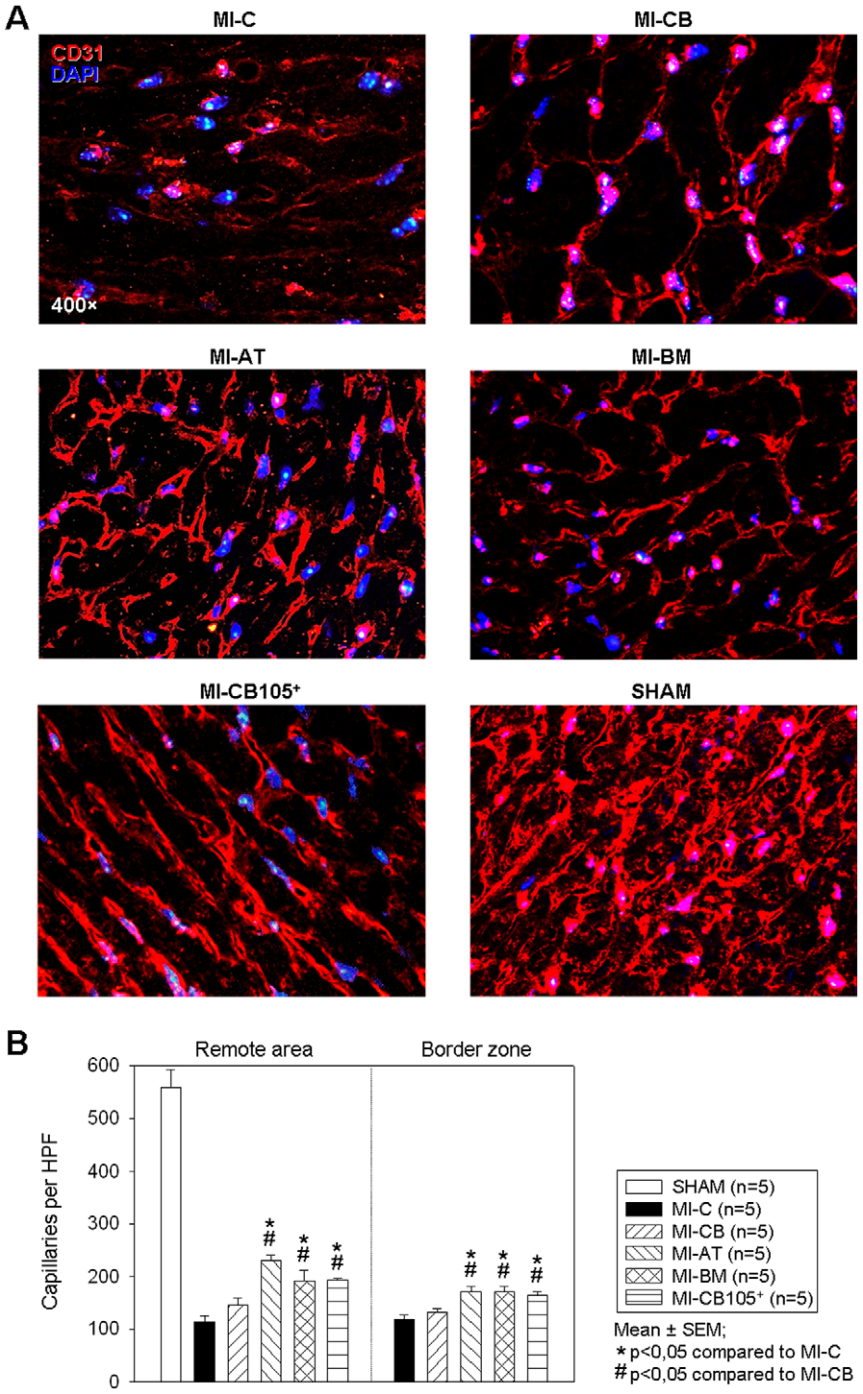
Capillary density 6 weeks after MI. ***A.*** Representative endothelial CD31 staining at the infarction border zone of level c sections. ***B.*** Capillary density in both the RA and the BZ of the LV is significantly higher in MI-AT, MI-BM and MI-CB105^+^ compared to MIC.

The capillary density was determined by CD31-staining 6 weeks after myocardial infarction. Examples of staining from the BZ (border zone) of the infarct area present a lower capillary density in MI-CB treated hearts and the MI-C group. (Figure 4A) Both, at the BZ as well as the RA (remote area), hearts implanted with AT-, BM- and CD105-purified CB-hMSC show a significant higher capillary density compared to hearts which have been treated with cells derived from CB (MI-CB) and the MI-C group, respectively (Figure 4B).

### Cardiac remodeling

**Figure 5:**
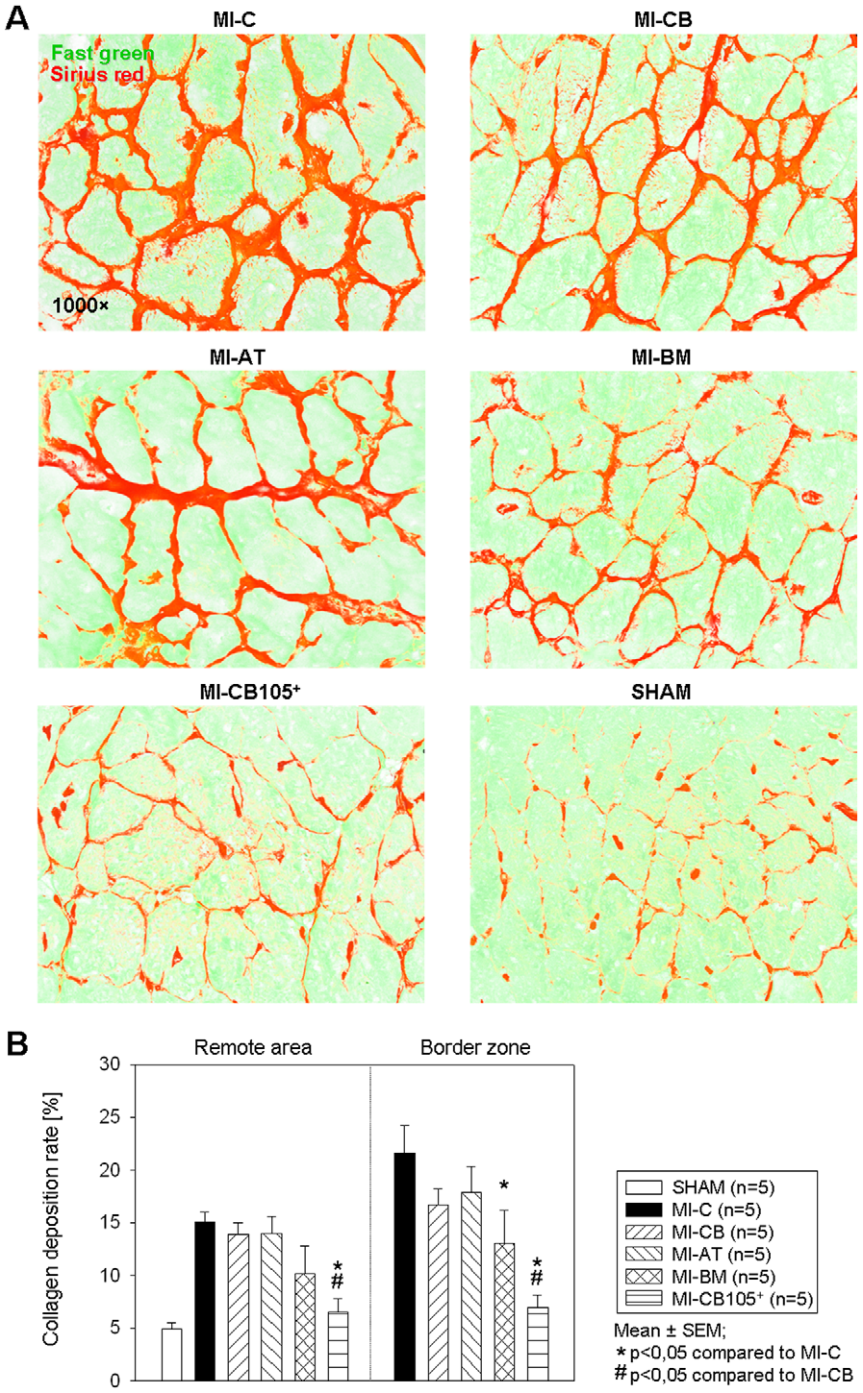
Fibrosis 6 weeks after MI. ***A.*** Representative Fast Green FCF (myocytes)/Sirius Red (fibrosis) stainings at the BZ. ***B.*** Significantly decrease of collagen deposition has been shown in both the RA and the BZ in MI-CB105^+^ compared to MI-CB and MI-C.

**Figure 6:**
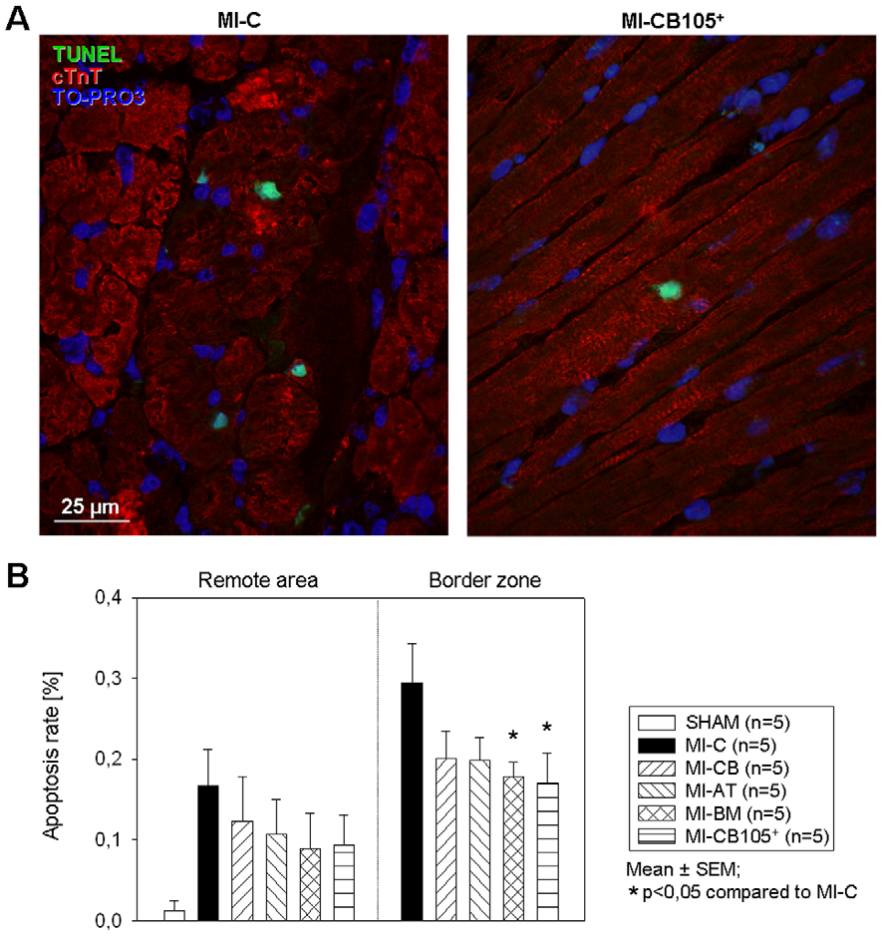
Late cardiomyocytes apoptosis. ***A.*** Representative immunostaining for TUNEL (green) and cardiac troponin (red) at the BZ 6 weeks after MI. ***B.*** Cardiomyocytes apoptosis was significantly reduced in the BZ in MI-BM and MI-CB105^+^ compared to MI-C.

Postinfarct cardiac remodeling serves as an important compensatory mechanism of congestive heart failure, characterized by progressive ventricular chamber dilatation, hypertrophy, fibrosis and prolonged cardiomyocyte apoptosis. Fibrosis resulted in extensive collagen deposition (Sirius red) and increased distance between myocytes (Fast green) 6 weeks after infarction. Figure 5A shows representative staining images from the BZ indicating a higher portion of collagen deposition in the MI-CB and the MI-C group. Hearts implanted with CD105-purified CB-hMSC showed a significant decrease of collagen deposition compared to MI-CB and the MI-C group, respectively in RA and in the BZ (Figure 5B). Figure 6A represents apoptotic nuclei of cardiomyocytes 6 weeks after myocardial infarction. A significantly reduced percentage of apoptotic cardiomyocytes could be found in the BZ of hearts implanted with CD105-purified CB-hMSC and BM-hMSC, respectively, compared to the MI-C group (Figure 6B).

### Engraftment and characterization of hMSC in infarcted murine hearts

**Figure 7:**
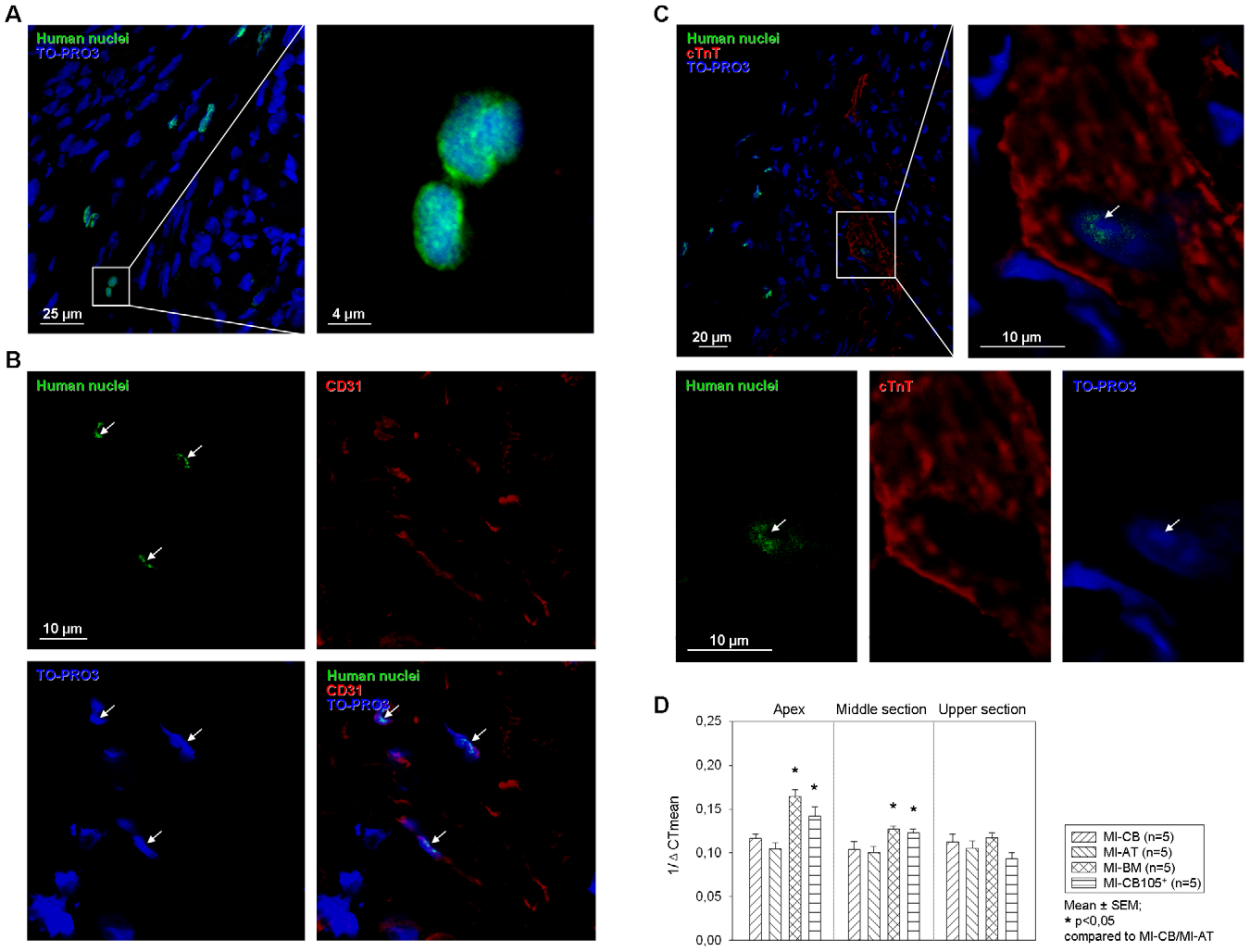
Identification of transplanted hMSC in infracted myocardium. 6 weeks after MI: ***A–C.*** Representative immunofluorescent micrographs of hearts transplanted with hMSC. ***A*** transplanted hMSC could be identified in infarcted myocardium ***B.*** A number of hMSC (Arrows, human nuclei in green) were co-localized with CD31 positive cells (red). ***C.*** Occasionally hMSC (Arrow, human nuclei in green) co-localized with cardiac troponin positive cell (red). (Confocal image, original magnification 630×) ***D.*** Quantitative real-time PCR analysis for human GAPDH expression level at different infarction sections: MI-BM and MI-CB105^+^ hearts show significantly higher localisation of human cells in the middle and apex section.

We evaluated human GAPDH expression at 3 different parts from the infarction area and identified implanted cells in the mouse tissue 6 weeks after myocardial infarction following hMSC application with selective binding human nuclei antibody (HNA) (Figure 7A). Double immunofluorescence staining with HNA and CD31 antibody revealed that at least some of the hMSC appeared to display endothelial cell-like phenotype (Figure 7B). Six weeks after cell transplantation, we observed a very low number of hMSC colocalized with cardiac Troponin T (cTnT) (Figure 7C). The frequency of cTnT-HNA double-positive cells from the engrafted stem cells was extremely low. There was no significant difference between different hMSC groups. It was not clear whether the transplanted cells had fused or differentiated into cardiomyocytes. Furthermore, higher human GAPDH expression was detected in the lower and the middle section of the infarcted hearts transplanted with BM-hMSC and CD105-purified CB derived hMSC in comparison to MI-AT and MI-CB (Figure 7D). There were no significant differences in the upper heart section.

### Real time acidification of viable hMSC *in vitro*

**Figure 8:**
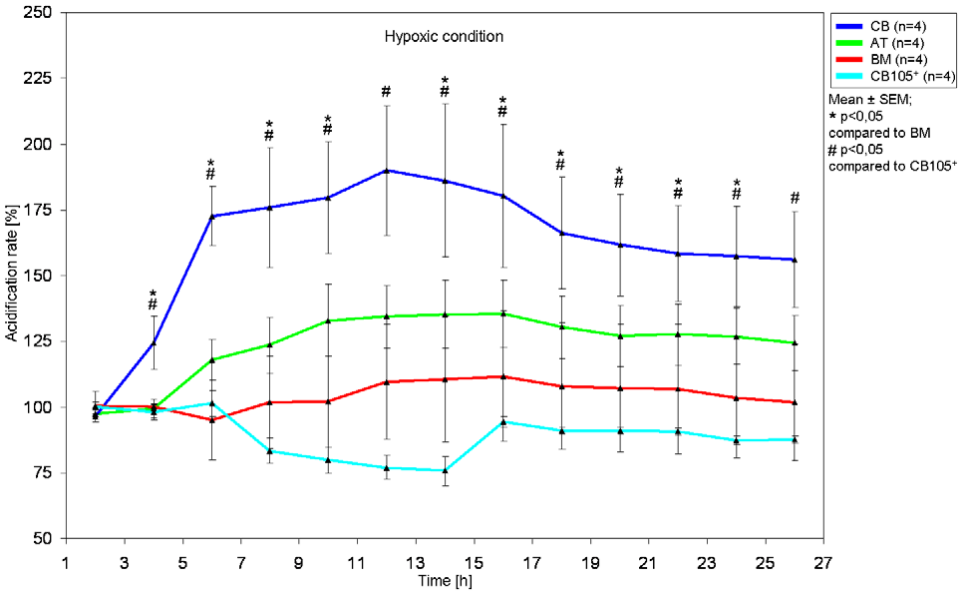
Real time acidification rate as a live cell parameter. CB derived hMSC show significant increased metabolic activity under hypoxic situation compared to BM- and CD105-purified CB-derived hMSC.

As the acidification is closely linked to the cellular energy metabolism, we measured the acidification rate of hMSC under normoxic as well as hypoxic conditions. While under normoxic conditions the cells showed no significant difference (data not shown), CB derived hMSC showed significantly higher metabolic activity than AT-, BM- and CD105-purified CB-hMSC under hypoxic conditions (Figure 8).

### Tube formation of hMSC *in vitro*

**Figure 9:**
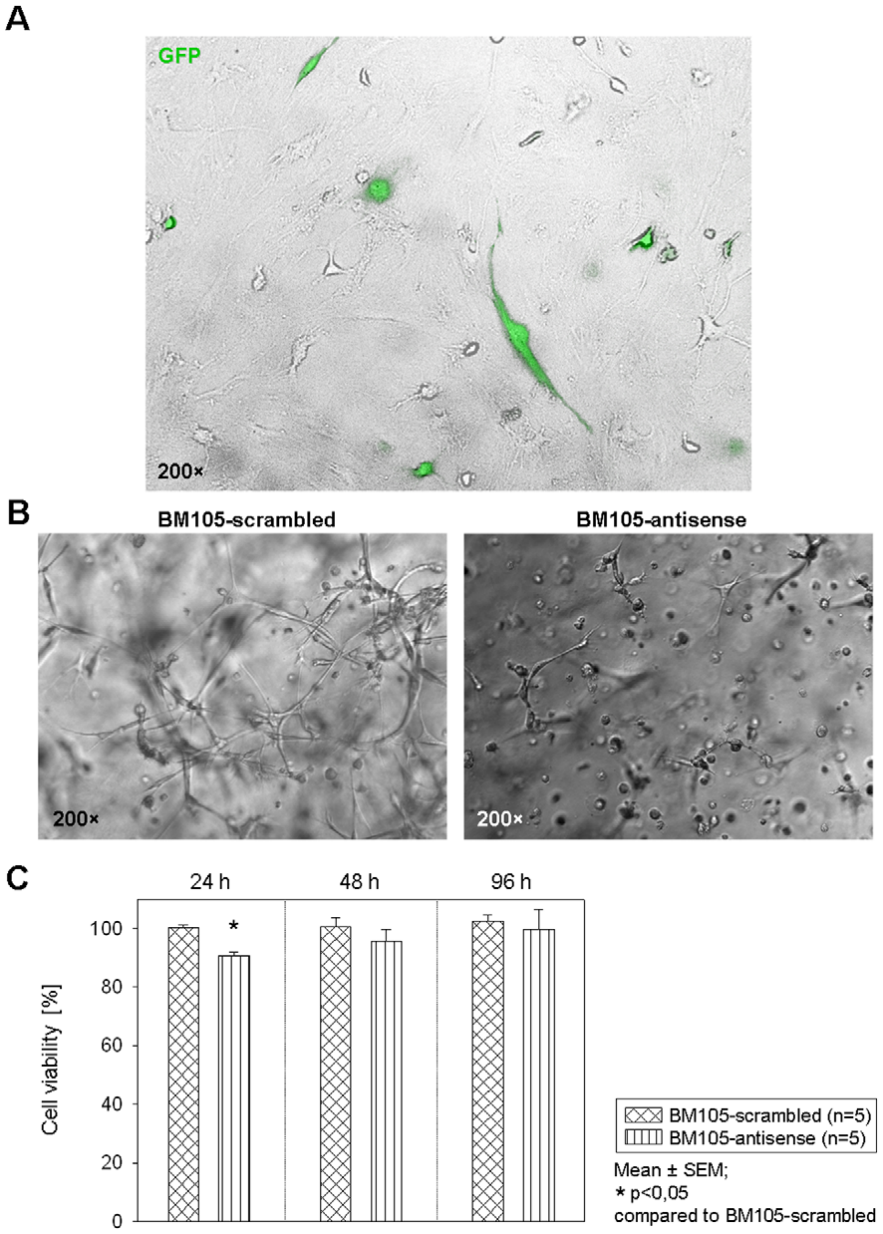
CD105 cell isolated from BM hMSC were transiently transfected with GFP, antisense-Oligodesoxynukleotide (ODN-CD105), scramble ODN. ***A.*** Transfection efficiency is shown on the upper picture (GFP) ***B.*** Silencing of CD105 blocks BM-hMSC tube formation compared to nature and scrambled group. The BM-hMSC were embedded in Matrigel and incubated in EGM-2 seven days after transfection and then imaged. *C.* Cell proliferation was analyzed at 24, 48 and 96 h after the ODN-CD105 and scramble ODN transfection by MTT assay.

To observe the influence of CD105 in the acceleration of network formation we compared nature BM derived hMSC with CD105 low BM-hMSC (antisense) using antisense phosphothiate-ODN. Figure 9 shows the delivery efficiency of antisense into hMSC and antisense delivery decreased network formation of BM-hMSC compared to nature and scrambled group.

### Cell proliferation of hMSC *in vitro*

The MTT assay is widely used to measure metabolic activity and cell proliferation. At different time points we detected metabolic activity of BM105-antisense and BM105-scrambled. After 24 h we observed a reduced viability for BM derived hMSC treated with BM105-antisense compared to scrambled group. The antisense blocking of CD105 seems to lose at later time points we measured (Figure 9C).

## Discussion

The present study for the first time systematically evaluated the cardiac regenerative capability of hMSC derived from different human origins in a *SCID* mouse left anterior descending (LAD) ligation model via intracardiac injection.

We showed that hMSC originating form different sources could induce significant morphological and functional differences in cardiac parameters. Infarcted hearts with hMSC-injection derived from BM displayed (I) a significant improvement in myocardial performance in comparison to those with hMSC-injection from AT and CB. Furthermore, BM-hMSC treated animals presented (II) a significantly reduced infarction area following diminished cardiac remodeling and (III) a better capillary density in the border zone of the MI. Significant higher localization of human cells could be seen in the middle and apex section of BM-hMSC treated hearts, which might be the result of a migration effect similar than that seen by cardiac stem cells [39]. BM-derived hMSC also showed (IV) the lowest metabolic activity in comparison to all other cells. In addition, the definitive application of a pure fraction of CD105^+^-hMSC from CB revealed overall a better myocardial performance than the whole proportion of CB derived hMSC and was similar to that of the MI-BM group.

hMSC are found in many adult tissues and represent an attractive stem cell pool due to their self renewing ability, high proliferative capacity and mesodermal differentiation potential [15]. For the isolation of hMSC, the BM displays one of the main sources next to alternative sites such as CB and AT [26], [28]. hMSC derived from different human sources are well characterized and share a consistent and reproducible gene expression profile [33], however they may behave differently with respect to morphology, expansion rate and differentiation potential under *in vitro* conditions [34], [40], [41]. It has been not determined if there might be therapeutic differences. In order to compare the differentiation potential of hMSC originating from CB, AT and BM, a MI model in the mouse was established as previously shown by our group [42]. Herein we are able to analyze structural, functional and molecular changes associated with acute MI.

Bieback and coworkers demonstrated that among different stem cell surface markers (CD29, 44, 73, 90, 105), CD105 was significantly lower expressed in CB-derived hMSC than in AT and BM [34]. Therefore, we have also analyzed a very pure fraction of CD105^+^-hMSC derived from human CB, which has been purified by an immunomagnetic isolation technique. CD105 or endoglin is a type I membrane glycoprotein, which is located on the cell surface and is also part of the TGF-β receptor complex [43]. Besides cytoskeletal organization, endoglin is also associated with the development of the cardiovascular system and vascular remodeling [43], [44]. Furthermore, it is a proliferation-associated and hypoxia-inducible protein which is efficiently expressed in endothelial cells during (tumor) angiogenesis [43], [45]–[47]. Hence, low or less CD105 might be a potential candidate for the overall worse performance of animals treated with the whole fraction of CB-derived hMSC. Additionally, the results of presented study show that after injection of the pure CD105^+^-fraction nearly equivalent values could be obtained as seen in the MI-BM-group. This might be due to the fact that CD105^+^-cells strongly activate the TGF-β1 receptor pathway which can then interact with downstream signaling to the Smad proteins, which seem to be involved in cardiac fibrosis and scar remodeling [48], [49]. The stem cell surface marker CD105 additionally might be of importance during the regeneration process of the infarcted heart. Herein it could be shown that CD105 prevents hypoxia-induced apoptosis in endothelial cells [50] and that downregulation of CD105 mRNA and protein expression resulted in a reduced inhibitory effect of TGF-β1 on cell proliferation, migration and microvessel formation [51]. Further studies with genetic manipulation of stem cells by virus vector [52], [53], nanoparticles [54], [55] or gene-activated matrix [56], [57] and genome-wide transcriptome profiling analysis [58] are necessary to elucidate the underlying mechanism by which CD105^+^-cells improve the therapeutic efficacy of cell transplantation in treating myocardium.

Taken together, the presented study demonstrated that hMSC display different regenerative effects in the post-infarct period. Especially for CB-derived hMSC, this might be due the fact of low CD105 purity. These results underscore the importance of a detailed evaluation of the different sources of hMSC prior to their clinical application, in order to increase the patient benefit of stem cell therapy after MI.

## Materials and Methods

### Culture of hMSC

For studies involving human tissues we obtained ethical approval of the local ethical committees, Medical Ethics Commission II, Medical Faculty Mannheim, Heidelberg University and Heidelberg University Ethical Board. The initial approvals 48/05 (Medical Ethic Commission II) regarding isolation of mesenchymal stem cells from umbilical cord blood and 2006-192N-MA (Medical Ethic Commission II) regarding isolation and characterisation of mesenchymal stem cells from adipose tissue have been reconfirmed on 26.02.2009. Bone marrow for research purposes was received according to the approval by the Heidelberg University Ethical Board; approval nos.: 251/2002 and S-076/2007. All samples were taken after written consent using guidelines approved by the Ethic Committee on the Use of Human Subjects at the University of Heidelberg.

According to requirements of the START-MSC-Project we used hMSC from the three different human sources cord blood (CB), bone marrow (BM), and adipose tissue (AT) which were isolated and prepared as previously described in detail [34]. We cultured all the hMSCs at 37°C at a humified atmosphere containing 5% CO_2_ and a mean cell density of 4±1×10^3^/cm^2^ in stem cell medium (MSCGM; Lonza, Walkersville, MD, USA) to 70–80% confluency. Cells were harvested at sub-confluency using trypsin. After the third passage, cells have been used for subsequent *in vitro* and *in vivo* experiments.

### CD105^+^ separation

To isolate CD105^+^ cells from human CB derived hMSC we used magnetic separation with CD105 MicroBeads following the instructions of MACS® (Miltenyi Biotec, Germany) using 20 µl MicroBeads per 250.000 augmented hMSC. The positive fraction was used for subsequent experimentation. A number of 10^7^ cells were resuspended in PBS with 2 mM EDTA and 0.5% bovine serum albumin and loaded into the separation columns after MicroBeads incubation. The positive fraction was used for in vivo experiments without further culture. In order to analyze the surface expression of CD105 by Flow Cytometry, magnetic separation was carried on at passage 3. Subsequently, CD105^+^ signal was monitored at passages 3, 4 and 5.

### Immuno phenotypic analysis

Cell surface antigen phenotyping of CB, AT and BM derived hMSC was performed at passage 3. CD105 expression level in CD105^+^ enriched CB-hMSC population was monitored at passages 3, 4 and 5. The following cell-surface epitopes were marked with the anti-human antibodies CD29-APC, CD44-PerCP-Cy5,5, CD45-V500, CD73-PE, CD90-PE (Becton Dickinson, Germany) and CD105-Alexa488 (AbD Serotec, UK). Mouse isotype antibodies served as control. 2×10^4^ labeled cells were acquired and analyzed using a FACS Scan flow cytometer (LSRII) running with CellQuest-Software (Becton Dickinson).

### Experimental design of the animal model

The federal animal care committee of LALLF Mecklenburg-Vorpommern (Germany) approved the study protocol (approval number LALLF M-V/TSD/7221.3-1.1-036/07). SCID mice (strain CB17/Icr-Prkdc-scid) were purchased from Charles River Laboratories (Sulzfeld, Germany). *SCID* mice (male, 20±1 g, Charles River Laboratories) were randomly assigned to 5 groups: Sham operation (Sham, n = 10) and 4 MI groups with implanted hMSC of the respective source (MI-CB n = 10, MI-CB105 n = 10, MI-AT n = 10, MI-BM n = 10). Infarcted animals treated with BD Matrigel™ Matrix alone served as controls (MI-C n = 10). A subset of randomly selected mice (n = 7) were assessed for functional measurement, histological and real time polymerase chain reaction (real time-PCR) evaluation at 6 weeks after LAD-ligation.

### Generation of MI in mice and stem cell implantation

Mice were anesthetized with tribrom ethanol (Avertin® 0,35 mg/kg, intraperitoneal). After thoracotomy and preparation, the left anterior descending coronary artery (LAD) was permanently ligated. Immediately after LAD-ligation, each mouse received an intramyocardial injection of 400.000 hMSC in BD Matrigel™ Matrix (BD Biosciences USA), or BD Matrigel™ Matrix alone for MI-C similar to a previous study [59]. Along the border of the blanched myocardium 4×5 µl injections were given. Sham operated mice underwent identical surgical procedures without LAD-ligation but followed by intramyocardial BD Matrigel™ Matrix injection without cells.

### Left ventricular catheterization

Six weeks after surgery, mice underwent pressure-volume (P/V) loop measurements according to the protocol of CardioDynamics BV (CD Leycom, Zoetermeer, Netherlands). Data were collected with the Millar Pressure-Volume System (Ultra-Miniature Pressure-Volume Catheter (model SPR-1030), Millar Pressure Conductance Unit (model MPCU-200) and Millar PowerLab data-acquisition hardware; emka Technologies, Paris, France). Calibration of pressure and volume was performed by equating the minimal and maximal conductances with minimal (0 mmHg) and maximal (100 mmHg) pressures as well as minimal and maximal blood volumes received from venous circulation. After inserting the catheter into the carotid artery, retrograde access to the left ventricle (LV) was achieved. P/V loops were recorded under normal conditions (baseline) followed by stress conditions mediated by intravenous dobutamine administration (10 µg/kg/min, Sigma-Aldrich, Deisenhofen, Germany). Volume signal was corrected by measurement of wall conductance (parallel volume) via hypertonic saline (5%) injection. Data were analyzed with IOX Version 1.8.3.20 software (emka Technologies). After P/V loop measurements, mice were euthanized. Hearts were arrested in diastole with potassium chloride. Each heart was removed, embedded in O.C.T.™ Compound (Tissue-Tek®; Zoeterwoude, Niederlande) and snap-frozen in liquid nitrogen. For histological and biomolecular investigations the infarct area of heart tissue has been divided into 4 horizontal levels from top to bottom within each given amount of 5 µm sections were cut. The three interlayers between the mentioned levels have been collected separately for RNA isolation.

### Infarction size and fibrosis analysis

Heart sections of 4 horizontal infarct levels (5 µm) were stained with Fast Green FCF (Sigma-Aldrich) and Sirius Red (Division Chroma, Münster, Germany). Two contiguous levels of the heart (n = 7 for each group) which represent the major infarct ratio were analyzed using computerized planimetry (Axio Vision LE Rel. 4.5 software; Zeiss, Jena, Germany). To evaluate fibrosis (n = 5 for each group), the sirius red positive regions of collagen deposition in the remote area (RA) near endocardial border were examined in 10 randomly chosen fields per section (one section per level; 630×) using computerized planimetry. Collagen density was expressed as the ratio of collagen deposition to myocardial tissue in percentage.

### Late cardiomyocytes apoptosis

To analyse cardiomyocyte apoptosis, heart sections of two contiguous levels of the heart (n = 5 for each group) which represent the major infarct ratio underwent terminal deoxynucleotidyl transferase-mediated dUTP nick end-labelling assay (DeadEnd Colorimetric TUNEL System; Promega; Madison, WI, USA) according to the manufacturer's instructions. Subsequently, slides were stained with rabbit polyclonal anti-troponin I primary antibody (Santa Cruz; Santa Cruz, USA) and goat anti-rabbit Alexa-Fluor® 568 (Molecular Probes™; Carlsbad, USA) conjugated secondary antibody, counterstained with TO-PRO®-3 iodide (Molecular Probes™) for nuclei and examined with confocal microscope. The number of TUNEL positive cardiomyocytes was counted in 10 randomly chosen HPFs (630×) per section (one section per level) for both RA and border zone (BZ; n = 5 for each group). Results were expressed as the proportion of the TUNEL positive cardiomyocytes nuclei to the total number of cardiomyocytes in percentage.

### Determination of capillary density

For immunohistological detection of capillaries, heart sections of two contiguous levels of the heart (n = 5 for each group) which represent the major infarct ratio were immunostained with polyclonal goat anti-CD31 (Santa Cruz) primary antibody followed by anti goat Alexa-Fluor® 568 (Molecular Probes™) conjugated secondary antibody and counterstained with DAPI. The sections were analyzed within the BZ and RA of the heart. Capillary density was assessed by counting the number of capillaries in 5 RA and 5 BZ randomly-chosen fields (400×). Results were expressed as capillaries per high power field (HPF).

### Human cell detection

For identification of implanted hMSC total RNA was isolated from the three separately collected interlayers of cryosectioned hearts (n = 7 for each group) following the instructions of the TRIzol® Reagent (Invitrogen; Carlsbad, USA). For reverse transcription of total RNA amount (2 µg) and first-strand synthesis of cDNA, SuperScript® III Reverse Transcriptase (Invitrogen) and oligo (dT)_15_ Primer (Promega) were applied. Quantitative real time-PCR was performed with StepOnePlus™ Real-Time PCR System (Applied Biosystems Foster City, CA, USA) in TaqMan® Universal Master Mix, No AmpErase® UNG (Applied Biosystems) according to the instructions of the manufacturer using the following program: 1 cycle of 50°C for 2 min, 1 cycle of 95°C for 10 min, and 40 cycles of 95°C for 15 s and 60°C for 1 min. Human GAPDH (TaqMan® Gene Expression Assay ID: Hs99999905_m1; Applied Biosystems) were tested in at least triplicate, normalized against mouse GAPDH (Endogenous Control: 4352339E) and negative controls were included in each assay. Cycle thresholds (C_T_) for single reactions were determined with StepOne™ Software 2.0 (formula: ΔC_T_  =  C_T hGAPDH_ - C_T mGAPDH_; Applied Biosystems). Resulting ΔC_T_ of triplicates was averaged and reciprocated to present the quantity of attend human cells. For immunohistological identification of human cells, heart sections were immunostained following the instructions of Vector® M.O.M.™ Immunodetection Kit (LINARIS; Wertheim-Bettingen, Germany) to localize mouse primary monoclonal and polyclonal antibodies on mouse tissue using monoclonal anti-human-nuclei (CHEMICON; Billerica, MA, USA) primary antibody followed by anti mouse Alexa-Fluor® 488 (Molecular Probes™) conjugated secondary antibody and counterstained with TO-PRO®-3 iodide (Molecular Probes™).

### Human cell differentiation potential

In order to investigate the differentiation capacity of hMSC after transplantation into the infarcted heart multiple antibodies staining was performed. Polyclonal goat anti-CD31 (Santa Cruz) primary antibody was initially applied to the section followed by anti goat Alexa-Fluor® 488 (Molecular Probes™) secondary antibody incubation. Subsequently, human nuclei were stained following the protocol previously described and a rabbit polyclonal anti-troponin I primary antibody (Santa Cruz) was applied to the sections. A goat anti-rabbit Alexa-Fluor® 568 (Molecular Probes™) was utilized during secondary antibody reaction. Counterstaining was achieved by TO-PRO®-3 iodide (Molecular Probes™) nuclear staining. The samples were analyzed using a LSM 780 confocal microscopy (Carl Zeiss, Jena).

### Real time acidification of viable hMSC *in vitro*

The silicon sensor chip technology allows the observation of cellular behaviour in cell cultures. Online monitoring was performed with the Bionas® 2500 analyzing system (Bionas, Rostock, Germany) [60], [61]. The cells were seeded in duplicate 24 h before measurement directly on the chip surface to assure highly specific signal detection. The cell concentration was adapted in such a way that the cells reach approximately 80% confluence on the sensor chip after 24 h. The measurement is noninvasive and label-free. The media flow over the cells is stopped periodically. Breakdown products (lactate, CO_2_) and the oxygen consumption of cells result in a change of pH and oxygen content in the medium. These changes are measured in the stop phases of the pump cycle. In the following pump phase “used” medium is exchanged for fresh medium. The stop and go cycle of 8 min each is carried out over the whole experiment. Acidification rates are calculated as the slope of changes in every stop phase related to the basal signal in %. The changes of pH are measured on the sensor chip by Ion Sensitive Field Effect Transistors (ISFETs). For measurement in the Bionas® 2500 analyzing system medium without bicarbonate buffer (running medium) with 1 mM HEPES, 0.1% FCS, 10.000 U penicillin and 10 mg streptomycin/ml was used. The pH of the running medium was adjusted to 7.4 and the osmolarity to 290 mOsm/kg. For measurement under hypoxic conditions the analyzing system was operating in a nitrogen environment. At the end of the experiment the cells were killed by addition of 0.2% Triton X-100 to the running medium to get a basic signal without living cells on the sensor surfaces (negative control).

### *In Vitro* Functional Differentiation Assay

To induce adipogenic differentiation, hMSC from different sources were seeded at a density of 3×10^3^ cells per cm^2^ and cultured for up to 3 weeks in cell culture medium supplemented with 10−8 M dexamethasone, 2.5 µg/ml insulin, and 100 µM indomethacin. To induce chondrogenic differentiation, 3×10^5^ hMSC were cultured in 1 ml of chondrogenic induction medium (cell culture medium supplemented with 0.1 µM dexamethasone, 1 mM sodium pyruvate, 0.17 mM l-ascorbic acid 2-phosphate, 0.35 mM l-proline, 6.25 μg/ml insulin, 6.25 μg/ml transferrin, 6.25 ng/ml selenite, 5.33 μg/ml linolic acid, 1.25 mg/ml bovine serum albumin, and 0.01 µg/ml transforming growth factor-β3) in the tip of a 15-ml conical tube to allow aggregation of the cells in suspension culture. The induction of chondrogenic differentiation was performed for 4 weeks. The differentiation capacity toward different cell lineages was verified by morphology changes and immunostaining for specific markers, that is, aggrecan for chondrocytes and, fatty acid binding protein (FABP-4) for adipocytes

### Antisense-Oligodesoxynukleotide (ODN) blockade

BM-hMSC at passage 3 was seeded 24 h before transfection. The cell concentration was adapted in such a way that the cells reach approximately 60% confluence on the well after 24 h. To reduce the expression of CD105 in BM derived hMSC phosphothiate-ODN with the antisense-sequence 5′-ATGCTGTCCACGTGGG-3′ (Eurogentec, Germany) was transfected into the cells by Lipofectamine 2000 (Invitrogen) as the manufacturer described. A scrambled-ODN with the nonsense sequence 5′-ACTCGTGCTACGGTGG-3′ (Eurogentec) was used as control.

### Tube forming assay

To observe the network formation potential of BM derived hMSC with reduced expression compared to nature cells, BM-hMSC were seeded in 24-well plates and transfected as described. After 24 h 7×10^4^ cells were cultured in 4-well plates with 200 µl BD Matrigel™ Matrix (BD Biosciences) at 37°C and a humified atmosphere containing 5% CO_2_. After 30 min 200 µl endothelial cell medium (EGM-2; Lonza) was applied to each well, cultured continuously and daily changed.

### MTT assay

Before detection of metabolic activity of BM105-antisense and BM105-scrambled with MTT (3-[4,5-Dimethyl-2-thiazolyl]-2,5-diphenyl-2H-tetrazolium bromide; Sigma-Aldrich, St. Louis, MO, USA) assay, BM-hMSC were precultured in 96-well plates and transfected as described. After 24, 48 and 96 h of incubation at 37°C MTT (5 mg/ml in PBS) was added into each well. After 4 h of incubation at 37°C, the medium was removed and the purple crystals were dissolved in 100 µl dimethylsulfoxide (DMSO). Absorbance was measured by a microplate reader (Model 680, Bio-Rad) at a wavelength of 550 nm with a reference wavelength of 655 nm. The results were expressed as the percentage of viable BM105-antisense and BM105-scrambled with respect to BM105-GFP. Cell viability was calculated using the following equation:

Cell Viability (%)  =  (OD550-OD655, samples)/(OD550-OD655, control) ×100%

### Statistical analysis

Statistical analysis was performed using SigmaStat 3.0 (Chicago, USA). Results are expressed as mean ± SEM. Overall comparisons of the treatment groups were performed by using the one-way analysis of variance (ANOVA) method that applies post-hoc multiple Holm-Sidak tests, and by using the nonparametric Kruskal-Wallis (failing normality) or post-hoc multiple Dunn tests. *P* values <0.05 were considered as statistically significant.
